# Seasonal variations of insect abundance: Correlating growth stage-specific metrics with weather patterns in Rangpur Region, Bangladesh

**DOI:** 10.1016/j.heliyon.2024.e38121

**Published:** 2024-09-19

**Authors:** Tapon Kumar Roy, Mir Md Moniruzzaman Kabir, Sanjida Akter, Abu Nayeem, Zakaria Alam, Md Rokebul Hasan, Md Nazmul Bari, Anamika Sannal

**Affiliations:** a Entomology Division, Bangladesh Rice Research Institute (BRRI), Gazipur, Bangladesh; b Tuber Crops Research Centre, Bangladesh Agriculture Research Institute, BARI, Gazipur, Bangladesh; c Plant Breeding Division, Bangladesh Rice Research Institute (BRRI), Gazipur, Bangladesh; d Plant Pathology Division, Bangabandhu Sheikh Mujibur Rahman Agricultural University (BSMRAU), Gazipur, Bangladesh

**Keywords:** Northern region, Correlation, Insect abundance, Weather parameter, Beneficial & harmful insects, Seasonal abundance

## Abstract

The disclosure of insect occurrences correlating with meteorological data is pivotal in devising effective management strategies for rice fields. The study collected data on harmful and beneficial insects, along with meteorological variables, for three consecutive rice-growing seasons in 2021 and 2022. Month-wise insect distribution were delineated, with prominent species such as green leafhopper, brown planthopper, white-backed planthopper, rice whorl maggot, damselfly, parasitic wasps and green mirid bug observed consistently. Notably, most of the harmful insects were predominantly present during March–July and September–October. The spatial distribution, further segmented based on the rice-growing seasons, revealing the Aus season's preeminence in hosting harmful insects followed by Aman and Boro. The prevalence of most of the harmful insects were in tillering stage. Beneficial insects, displayed their dominance during specific months, growth stage, emphasizing their potential role in controlling harmful species and their prevalence were increased by increased of harmful insects. Furthermore, the study elucidated the correlation between climatic factors and insect abundance, emphasizing the role of temperature, rainfall and relative humidity. Temperature metrics and relative humidity manifested significant associations with several insects both harmful and beneficial species differently. While rainfall notably correlated with rice bug, short-horned grasshoppers, yellow stem borer, and non-significant association with other insects. The study underscored mutual dependencies between predator and prey species, emphasizing the ecological balance within the agricultural ecosystem. This comprehensive analysis indicated the need for integrated pest management strategies, considering both harmful and beneficial insect dynamics, to promote sustainable practices for optimizing rice production in changing climatic scenarios.

## Highlights

• Highest prevalence of insects was found in Aus season, which was followed by T Aman and Boro season.
• Insects like GLH, BPH, WBPH (Harmful), and damselflies, PW, GMB (Beneficial) were the dominant species throughout the year.
• Rising temperatures, coupled with other weather factors, accelerate insect activity and growth.
• Significant correlation observed between beneficial and harmful insects.

## Graphical abstract

**Figure:**
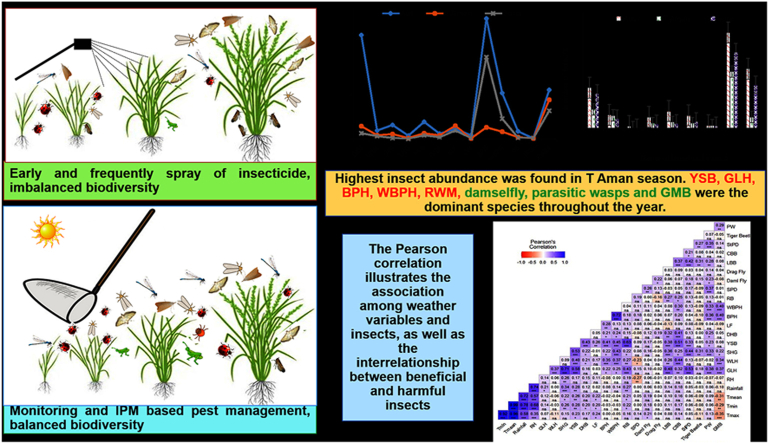

## 1 Introduction

Rice (*Oryza sativa* L.; Family: Poaceae) stands as one of the foremost cultivated cereals and holds global significance as a globally important food crops. For food security, rice plays very crucial role and is the main staple food in Bangladesh. In Bangladesh, the assurance of food security is synonymous with ensuring rice security [1]. It is grown extensively throughout the country. Rice is cultivated in wetland areas, where other crops struggle to thrive due to waterlogging conditions. There are mainly three rice growing seasons in Bangladesh i.e Aus (monsoon rice), Aman (rain-fed with supplemental irrigation) and Boro (irrigated rice). In Bangladesh, rice cultivation occurs year-round, with cultivation efforts intensifying progressively to satisfy the escalating demands of a growing population. Notably, during the 2021-22 rice cultivation season encompassing Aus, Aman, and Boro, the country achieved a significant rice yield of 38.15 million tons, cultivated across a vast expanse of 11.69 million hectares [2]. Among 11.69 million hectares the Aus, Aman and Boro cultivated area of Bangladesh in 2021-22 was 1.19, 5.68 and 4.79 million hectare and the production was 3.00, 14.56, 20.19 million tons respectively [2]. But, the production of rice depends on various factors like variety, cultural practices and biotic and abiotic environmental conditions [3,4]. Throughout the process of rice cultivation, insects commonly inhabit in the rice fields. Their presence can lead to production losses directly and indirectly at various stages of the crop's growth, from its early to mature stage [5]. However, the prevalence and intensity of these infestations may fluctuate, potentially influenced by changes in climate and evolving cropping practices in contemporary rice farming. So, rice insect pest is a major problem for rice production in rice growing areas including Bangladesh. The warm and humid climate of Bangladesh is conductive to the proliferation of insect pests. The three rice crops grown, under diverse ecological conditions are attacked throughout the growing periods by a number of insect pests. Within the rice ecosystem of Bangladesh, there exists a diverse assembly of 232 insect pests specific to rice cultivation, alongside 375 species of beneficial arthropod species including predator and parasitoids [6,7]. Interestingly, while fewer than twenty species causing notable yield reductions in India, but Bangladesh confronts a broader spectrum ranging from 20 to 33 species that are deemed significant contributors to economic losses in rice cultivation [8,9]. Furthermore, rice insect categorizations into major and minor pests and notable major pests in Bangladesh include the brown planthopper (*Nilaparvata lugens)*, stem borer (*Scirpohaga incertula)*, rice leaffolders (*Cnaphalocrocis medinalis)*, green leafhopper (*Nephotettix virescens)*, rice hispa (*Dicladispa armigera)*, gall midge (*Orseolia oryzae*), and white-backed planthopper (*Sogatella furcifera)*, as highlighted in a recent study [7,10]. Pests like green leafhopper, brown planthoppers, and white backed planthoppers causes significant damage to rice crops by suck the sap, weakening plants, reducing grain yield, and spreading harmful pathogens [11]. Moreover, new insects are introduced in rice crops, used rice plant as host like Fall Armyworm (*Spodoptera frugiperda)* [12].

Based on different studies, under severe infestation scenarios, the loss to rice yields attributable to stem borer damage has been estimated between 20 % and 70 % [[13], [14], [15]]. Similarly, during leaffolder outbreaks, yield reductions can range from 30 % to 80 % [16], and rice bug infestations have been linked to grain yield declines of 50 %–87 % [17]. Rice hispa and brown planthopper are also notable contributors to yield reduction, approximately 62 % and 44 % reductions, respectively [10]. Broadly, insect pests during rice cultivation in Bangladesh lead to an average yield decrement of about 18 %, with the predominant reliance on synthetic insecticides for their control [9,18]. The impact of insect pests on both yield quantity and quality remains profound. Even in farming systems employing intensive pesticide applications and crops engineered with insect and disease resistance, yield losses are still substantial, estimated at between 20 % and 30 % of the overall production [19]. The magnitude of damage varies in different growth stage, seasons, years, locations and meteorological parameters [20]. Insect pest management has frequently hinged on the widespread application of synthetic insecticides, which inadvertently harms beneficial insects, exhibits resistance to other harmful insects, and cause environmental pollution and health hazards [21,22]. Other hand, global warming and climate changes alters insect behavior [23]. Understanding how insects react to climatic variable such as temperature, humidity, and rainfall variations is crucial. Climate change can elevate minor pests to major threats and demote once-major pests, reshaping agricultural challenges. Climate change is transmute insect behaviors and their presence, as seen in trends like more annual generations, higher survival rates, and earlier insect appearances [24]. This evolving climate change is also notably altering the status of insect pests [25]. Specific knowledge to understanding the abundance and population trends of pests is crucial for ensuring timely interventions and mitigating potential crop damages [24]. As apprehensions escalate over the detrimental effects of insecticides and changing climatic conditions on both sustainable farming practices and human health, there has been an impetus towards the adoption of integrated pest management (IPM) methodologies [26]. This holistic strategy encompasses the vigilant surveillance and predictive analysis of pest populations within agricultural fields, guiding optimal timing for insecticide application. Such precision aims to curtail crop losses, enhance pest management efficacy, and optimize production costs.

Emphasizing the significance of surveying and monitoring insect pest species within ecosystems, it becomes evident that such efforts are pivotal for crafting integrated pest management strategies. The entomologists of Bangladesh Rice Research Institute (BRRI) conducted extensive surveys on rice insect pests across various agro-ecological regions, encompassing diverse rice varieties, cultivation periods, and growth phases [27]. However, there also need to understand the abundance and diversity of rice insect pests in different region. But it is very difficult to performed the task at a time in all region. Therefore, the aim of the present study is to prepare a list of rice field insect pests, natural enemies and their abundance on different stages of rice plants and in different growing seasons, and their variation in climate change situation in northern region, Bangladesh.

## 2 Materials and METHODS

### 2.1 Survey site

**Fig. 1:**
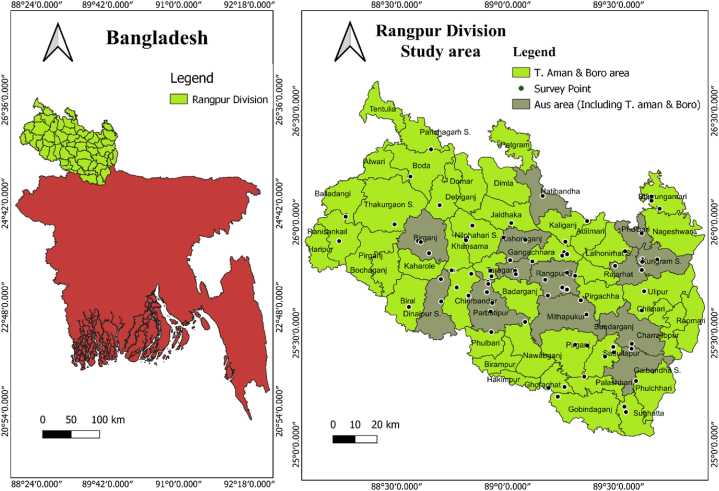
Study area of collecting insects through hand sweeping in Rangpur division in Aus, T. Aman & Boro season of 2021 and 2022.

The survey was conducted in various locations of Rangpur division (AEZ 1, 2 and 3 including Thakurgaon, Dinajpur, Lalmonirhat, Kurigram, Nilphamari, Gaibandha, Rangpur district) in Aus, T Aman and Boro season during 2021 and 2022. Survey was conducted in six upazila of Gaibandha, five upazila of Rangpur, 3 upazila of Nilphamari, 3 upazilla of Lalmonirhat, 5 upazila of Kurigram, 8 upazila of Dinajpur, 3 upazila of Panchagarh and 2 upazila of Thakurgaon district. The study areas were presented in Fig. 1.

### 2.2 Collection of insects

Harmful insects and natural enemies was collected by hand sweeping in Aus, Aman and Boro seasons and four growing stage i.e., seedling, active tillering, maximum tillering and booting to heading stage (Table 1) from 2021 to 2022 respectively. Twenty complete sweep was done randomly in one spot and then repeated for three times besides 1st complete area. One time complete sweep in a spot was considered as a replication. The distance among the spots at least fifteen km from each other and three rice fields at each spot for each season was selected as replications. Sweeping was conducted at various levels within the plant canopy including the interspaces between plants and extending as close to the base of the plants as possible. Sweeping was done for collection of insects and their natural enemies within 8.30am–12.00pm. The diameter of sweep net was 12 inches but in seedling stage rectangular sweep net developed by Ref. [28] was used with a handle. Weather data was collected from weather station of BRRI regional station Rangpur and presented in Table 2.

**Table 1:** Month wise growth phrase of rice plants in Rangpur, Division of Bangladesh.

SL	Growth phase	Aus	Aman	Boro
1	Seedling	March–April	July–August	December–January
2	Active tillering	April–May	August–September	January–February
3	Maximum tillering	May–June	September–October	February–March
4	Booting to heading	June–July	September–October	March–April

**Table 2:** Weather information of the study area from January 2021 to December 2022.

Month	Tmax (^0^C)	Tmin (^0^C)	Tmean (^0^C)	Rain fall (mm)	RH (%)
January	22.15	11.39	16.77	0.121	78.62
February	24.91	12.03	18.47	1.268	69.67
March	30.33	18.12	24.22	0	65.1
April	30.27	21.47	25.87	4.212	77.05
May	30.37	23.02	26.7	9.063	79.01
June	30.52	24.83	27.68	10.74	82.28
July	31.88	26.28	29.08	6.637	79.24
August	31.77	26.37	29.07	8.824	80.81
September	31.43	25.37	28.4	5.348	81.8
October	30.70	22.9	26.8	5.29	78.27
November	28.46	16.18	22.32	0.033	69.23
December	25.42	13.4	19.41	0.065	71.27

### 2.3 Identification

After collection of insects and natural enemies, immediately placed a net bag. After then, taxonomic characters was determined by sorting, identifying and counting through naked eye, magnifying glass and help of microscope. Determination of taxonomic characters of colleting insects were done as per [29].

Shannon Weiner diversity index,

$$H^{\prime }=-\sum_{i=1}^{S}\left(Pi.\ln\left(Pi\right)\right)$$

(Where, H is the Shannon-Wiener diversity index.

S is the total number of species in the community.

Pi is the proportion of individuals belonging to the ith species relative to the total number of individuals.)

Maximum potential diversity: H_max_ = ln(S)

(Where, H_max_ is the maximum diversity.

ln is the natural logarithm.

S is the total number of species in the community.

$${\text{Pielou}}^{\prime }s\;\text{Evenness}\;\text{Index}\;\left(E\right):E=\frac{H}{\ln\;\left(S\right)}$$

(Where, E is Pielou's Evenness Index.

H is the Shannon-Wiener diversity index.

S is the total number of species.)

### 2.4 Data analysis

For performing ANOVA “doebioresearch” package was used in R studio software. We used “ggplot2” and “metan” package to perform correlation plot in R studio (versions 2023.09.0 + 463). Study area map was created by using QGIS software (versions 3.22.9). Data preparation and executed all statistical analyses of the survey data was performed by utilizing Microsoft Office Excel (2016).

## 3 Results

### 3.1 Comparative diversity of rice insects and natural enemies in study area

The diversity of harmful insects and their natural enemies was presented in Table 3. In our study of Rangpur division, the Shannon-Wiener diversity index (H′) was calculated as 2.493 (‘-’sign neglected), indicating a diverse community of harmful and beneficial insects. The value was approximately 81 % of the maximum potential diversity (Hmax = 3.091), suggesting that the community is operating at a relatively high level of diversity. The evenness of species (E) was calculated as 0.807 (‘-’sign neglected), signifying a balanced distribution of individuals among different species.

**Table 3:** Population dynamics and calculation of Shannon-Wiener diversity index, H_max_ and evenness of species.

Common name	Scientific Name	Order name	Pi	ln (pi)		Pi∗ln(pi)
**Harmful Insects** rowhead						
Green leafhopper	*Nephotettix virescens*	Hemiptera	0.104	−2.264		−0.235
White leafhopper	*Cofana spectra*	Homoptera	0.016	−4.118		−0.067
Short horned grasshopper	*Oxya* spp	Orthoptera	0.020	−3.934		−0.077
Long horned grasshopper	*Conocephalus longipennis*	Orthoptera	0.004	−5.494		−0.023
Yellow stem borer	*Scirpophaga incertulas*	Lepidoptera	0.027	−3.607		−0.098
Dark headed borer	*Chilo polychrysus*	Lepidoptera	0.012	−4.383		−0.055
Rice leaffolder	*Cnaphalocrocis medinalis*	Lepidoptera	0.041	−3.192		−0.131
Case worm	*Parapoynx stagnalis*	Lepidoptera	0.005	−5.279		−0.027
Brown planthopper	*Nilaparvata lugens*	Hemiptera	0.211	−1.556		−0.328
White backed planthopper	*Sogatella furcifera*	Hemiptera	0.071	−2.640		−0.188
Rice bug	*Leptocorisa oratoria*	Hemiptera	0.011	−4.554		−0.048
Rice Skipper	*Pelopidas mathias*	Lepidoptera	0.002	−6.074		−0.014
Rice whorl maggot	*Hydrellia philippina*	Diptera	0.139	−1.975		−0.274
**Natural Enemy** rowhead						
Spider	*Atypena formosana*	Araneae	0.057	−2.861		−0.164
	*Oxyopes javanus*					
	*Tetragnatha virescens*					
	*Argiope catenulata*					
Damsel fly	*Chrysoperla* spp	Odonata	0.024	−3.737		−0.089
Dragon fly	*Diplacodes* spp	Odonata	0.002	−6.074		−0.014
Ladybird beetle	Coccinella septempunctata	Coleoptera	0.012	−4.396		−0.054
Carabid beetle	*Ophionea nigrofasciata*	Coleoptera	0.016	−4.149		−0.065
Styphylinid beetle	Paederus fuscipes	Coleoptera	0.014	−4.236		−0.061
Tiger Beetle	Cicindella sexpunctata	Coleoptera	0.006	−5.187		−0.029
Parasitic wasp	*Apanteles cypris*	Hymenoptera	0.142	−1.953		−0.277
	*Tropobracon luteus*					
	*Xanthopimla flavolineata*					
	*Telenomus* sp					
Green mirid bug	*Cyrtorhinus lividipennis*	Hemiptera	0.063	−2.765	−0.174	
			H_max_ = 3.09	E = 0.807	H′ = 2.493	

### 3.2 Monthly distribution of harmful and beneficial insects

Month-wise distribution of harmful insects has been presented in Table 4. Among the harmful insects, green leafhopper (GLH), brown planthopper (BPH), white backed plant hopper (WBPH), and rice whorl maggot (RWM) were dominant throughout the year. Notably, GLH, short-horned grasshopper (SHG), long-horned grasshopper (LHG), caseworm (CW), brown plant hopper (BPH), white backed plant hopper (WBPH) and rice bug (RB) were showed highest prevalent and statistically significant difference in June. In contrast, BPH and WBPH followed higher prevalence from March to July and again in December. The abundance of white leafhopper (WLH) was statically peaked in January, and December followed by July. Yellow stem borer (YSB) exhibited greater abundance from May to July and then from September to October but significantly highest in June then followed by July. On the other hand, dark headed borer (DHB) was found statically significant and highest abundance in February, July and from September to October. Furthermore, rice leaffolder (RLF) prevalence was highest and statically significant variation in September, followed by June. The RWM showed highest and statically significant difference in July and December followed by June.

**Table 4:** Month wise abundance of harmful insects/20 complete sweep.

Month	GLH	WLH	SHG	LHG	YSB	DHB	RLF	CW	BPH	WBPH	RB	Rice skipper	RWM
January	4.00 d	2.50 a	0.75e	0.00d	0.26h	0.26e	0.51g	0.00g	6.75e	2.75f	0.00f	0.00c	7.00ef
February	1.67 g	1.00 c	0.33f	0.00d	1.01f	1.0 ab	0.67g	1.00b	2.00g	2.00g	0.00f	2.00a	6.33f
March	0.50 h	0.38 e	0.13gh	0.00d	1.25de	0.52 d	3.0 bc	0.25de	23.38b	3.75e	0.25e	0.00c	7.50e
April	0.49 h	0.26ef	0.16g	0.00d	0.67g	0.33e	2.42d	0.25de	13.43d	4.53d	0.30e	0.00c	4.67g
May	4.38 d	1.00 c	0.63e	0.00d	1.50c	0.25e	1.13f	0.38c	34.75a	7.00c	0.50c	0.00c	9.38d
June	25.71a	0.71 d	4.17a	1.43a	3.88 a	0.91bc	3.15b	1.14a	35.14a	15.43a	2.29a	0.14c	12.29b
July	17.0 b	2.00 b	2.44b	0.27bc	3.20 b	1.07a	1.93e	0.33cd	16.47c	4.67d	1.20b	0.40b	15.00a
August	2.47 f	1.27 c	1.27c	0.20cd	1.07ef	0.80c	2.0e	0.13f	2.53g	2.33 fg	0.40d	0.07c	10.40c
September	3.15 e	0.81cd	0.90d	0.53b	1.57c	1.05 ab	4.19a	0.24e	2.30g	1.14h	0.57c	0.10c	4.76g
October	3.20 e	0.00 f	1.00d	0.40bc	1.40cd	1.0 ab	2.80c	0.00g	3.40f	2.40 fg	0.40d	0.40b	2.00h
December	6.50 c	2.50 a	0.00h	0.00d	0.00f	0.00f	0.00h	0.00g	14.00d	14.00b	0.00f	0.00c	15.50a
Level of Significance	∗∗∗	∗∗∗	∗∗∗	∗∗∗	∗∗∗	∗∗∗	∗∗∗	∗∗∗	∗∗∗	∗∗∗	∗∗∗	∗∗∗	∗∗∗
CV	5.46	15.39	7.26	60.67	7.85	13.71	12.85	15.07	2.83	5.31	9.80	51.71	4.96
LSD	0.58	0.29	0.13	0.26	0.15	0.15	0.42	0.08	0.67	0.49	0.09	0.25	0.72

**Footnote**, GLH: Green leafhopper; WLH: White leafhopper; SHG: Short-horned grasshopper; LHG: Long horned grasshopper; YSB: Yellow stem borer; DHB: Dark headed borer; RLF: Rice Leaffolder; CW: Caseworm, BPH: Brown planthopper; WBPH: White-backed planthopper; RB: Rice bug; RWM: Rice whorl maggot.

Among the beneficial insects, the abundance of damselfly, parasitic wasps (PW), and green mirid bug (GMB) was dominant throughout the year (Table 5). The prevalence of spider (SPD) was highest and statically significant in March followed by June, April and July respectively. The abundance of damselfly were found in year round, peaked in March–April and from June to October but statically significant and highest in April, July and October. Statically highest prevalence of dragon fly was observed in October and December followed by June. Statically significant and highest abundance of ladybird beetle (LBB) was observed in July–August and followed by April, June and October. Significantly the prevalence of carabid beetle (CBB) and styphylinid beetle (STPD) were peaked in June and followed by July. While the highest abundance of tiger beetles was found in February. In contrast, significantly the highest prevalence of GMB was t in December followed by January.

**Table 5:** Month wise abundance of beneficial insects/20 complete sweep.

Month	SPD	Dam. fly	Drag. fly	LBB	CBB	STPD	Tiger beetle	PW	GMB
January	1.26 i	0.25 d	0.00 d	0.75 cd	0.25 h	0.50 de	0.25 e	4.75 I	13.75 b
February	1.67 h	0.67 cd	0.00 d	0.33 f	1.00 d	1.33 b	2.00 a	5.33 h	3.33 d
March	7.01 a	1.38 b	0.11 bcd	0.51 e	0.75 e	0.75 c	0.00 f	10.49 b	3.00 de
April	4.89 c	1.89 a	0.08 cd	0.92 b	0.58 f	1.17 b	0.25 e	10.58 b	2.58 e
May	3.28 e	0.75 c	0.00 d	0.66 d	1.13 c	0.25 f	0.00 f	9.85 c	7.50 c
June	6.37 b	1.57 ab	0.29 b	0.87 bc	1.87 a	1.74 a	0.43 c	12.31 a	7.29 c
July	3.73 d	1.87 a	0.20 bc	1.07 a	1.73 b	1.33 b	0.47 c	9.80 c	3.47 d
August	2.60 f	1.40 b	0.13 bcd	1.13 a	0.60 f	0.67 cd	0.27 de	5.87 g	1.20 f
September	1.95 g	1.24 b	0.05 cd	0.38 ef	0.95 d	0.76 c	0.33 d	7.52 e	1.52 f
October	3.20 e	1.60 ab	0.60 a	0.80 bc	0.40 g	0.40 ef	0.60 b	9.20 d	1.60 f
December	0.00 j	0.50 cd	0.50 a	0.00 g	0.00 i	0.00 g	0.00 f	6.50 f	21.00 a
Level of Significance	∗∗∗	∗∗∗	∗∗∗	∗∗∗	∗∗∗	∗∗∗	∗∗∗	∗∗∗	∗∗∗
CV	3.22	22.72	58.60	11.24	8.57	14.35	10.37	2.60	5.67
LSD	0.18	0.46	0.17	0.13	0.12	0.19	0.07	0.37	0.58

**Footnote**, SPD: Spider; Dam fly: Damsel fly; Drag fly: Dragon fly; LBB: Ladybird beetle; CBB: Carabid beetle; STPD: Styphylinid beetle; PW: Parasitic wasps; GMB: green mirid bug.

### 3.3 Seasonal distribution of harmful and beneficial insects

Among three rice growing season of Rangpur division, Bangladesh, the highest number of harmful insects were observed in Aus season. Significantly the highest abundance of GLH, WLH, SHG, LHG, YSB, DHB, CW, BPH, WBPH, RB and RWM was in Aus season followed by T. Aman and Boro season respectively except WLH, CW, RB (Table 6). The highest abundance of rice leaf folder (RLF) was observed in T. Aman season and which was statically significant and followed by Aus season. On the other-hand, spider, styphylinid beetle, parasitic wasp and green mirid bug were observed significantly highest in Aus season followed by Boro and T. Aman season respectively. Whereas damsel fly showed stastically highest in Aus & T. Aman season followed by Boro season. Dragon fly, ladybird beetle were showed statically significant and highest variation in Aus season followed by T. Aman & Boro season. The abundance of carabid beetle was observed statistically highest in Aus season which was followed by T Aman and Boro season respectively (Table 7).

**Table 6:** Seasonal distribution of harmful insects/20 complete sweep.

Season	GLH	WLH	SHG	LHG	YSB	DHB	RLF	CW	BPH	WBPH	RB	Rice skipper	RWM
Aus	23.79a	1.95 a	3.16 a	0.74 a	3.89 a	1.21 a	2.32 b	0.84 a	27.58a	10.47a	1.79 a	0.16 ab	11.26a
T. Aman	2.89 b	0.81 b	1.11 b	0.23 b	1.34 b	0.85 b	2.98 a	0.15 b	2.62 c	1.64 c	0.43 b	0.11 b	8.92b
Boro	1.29 c	0.71 b	0.21 c	0.00 c	0.82 c	0.38 c	1.94 c	0.24 b	18.74b	4.62 b	0.29 b	0.18 a	6.45c
Level of Significance	∗∗∗	∗∗∗	∗∗∗	∗∗∗	∗∗∗	∗∗∗	∗∗∗	∗∗∗	∗∗∗	∗∗∗	∗∗∗	∗	∗∗∗
CV	1.16	8.11	2.71	9.62	2.53	10.09	3.24	14.15	0.58	1.62	10.73	17.21	0.54
LSD	0.22	0.19	0.08	0.06	1.11	0.16	0.16	0.12	0.19	0.18	0.18	0.05	0.09

Footnote, GLH: Green leafhopper; WLH: White leafhopper; SHG: Short-horned grasshopper; LHG: Long horned grasshopper; YSB: Yellow stem borer; DHB: Dark headed borer; RLF: Rice Leaffolder; CW: Caseworm, BPH: Brown planthopper; WBPH: White-backed planthopper; RB: Rice bug; RWM: Rice whorl maggot.

**Table 7:** Seasonal distribution of beneficial insect/20 complete sweeps.

Season	SPD	Dam. fly	Drag. fly	LBB	CBB	STPD	Tiger Beetle	PW	GMB
Aus	4.94 a	1.63 a	0.26 a	1.37 a	2.11 a	1.57 a	0.37	11.68 a	7.26 a
T. Aman	2.32 c	1.57 a	0.13 b	0.64 b	0.80 b	0.64 c	0.36	6.91 c	1.38 c
Boro	4.26 b	1.18 b	0.09 b	0.56 b	0.59 c	0.82 b	0.29	9.29 b	5.29 b
Level of Significance	∗∗∗	∗∗∗	∗∗∗	∗∗∗	∗∗∗	∗∗∗	ns	∗∗	∗∗∗
CV	1.07	3.87	17.67	6.10	5.26	5.27	12.26	0.78	0.91
LSD	0.08	0.11	0.06	0.10	0.12	0.11	–	0.14	0.08

**Footnote**, SPD: Spider; Dam fly: Damsel fly; Drag fly: Dragon fly; LBB: Ladybird beetle; CBB: Carabid beetle; STPD: Styphylinid beetle; PW: Parasitic wasps; GMB: green mirid bug.

### 3.4 Abundance of harmful and beneficial insects according to growth stage

Among different harmful insects, the prevalence of GLH was significantly highest at active tillering stage followed by seedling and maximum tillering stage respectively (Table 8). Conversely, the prevalence of whorl maggot and WLH were statistically highest in seedling stage followed by active tillering stage, maximum tillering stage and booting to heading stages, respectively. Significantly the highest abundance of SHG was observed in active tillering and seedling stage followed by maximum tillering and booting stage respectively. Other-hand, significantly the highest prevalence of YSB and rice skipper were found in active tillering stage followed by maximum tillering and booting stage. Highest abundance of DHB was observed in active tillering stage followed by maximum tillering and booting stage respectively. Statically highest number of BPH was found in maximum tillering stage followed by active tillering stage and WBPH was active tillering stage and seedling stage followed by maximum tillering stage. RB was found statically highest at maximum and booting to heading stage followed by active tillering stage. The prevalence of beneficial insects varied across different growth stages. Green mirid bug (GMB) was found to be dominant and statically significant during the seedling stage, followed by the active tillering, maximum tillering, and booting to heading stages, respectively. Damsel fly, and CBB, were showed statically highest prevalence during the active tillering and maximum tillering stage followed by booting stage (Table 9). Conversely, the prevalence of spiders was statically highest in the active tillering, maximum tillering and booting stage, followed by seedling stages. The prevalence of LBB and tiger beetle were statically highest at active tillering followed by maximum tillering stage. STPD was significantly found highest at active tillering and booting stage followed by maximum tilering stage. The abundance of PW was significantly highest at active tillering stage followed by maximum tillering and booting stage.

**Table 8:** Growth stage wise abundance of harmful insects/20 complete sweeps.

Growth Stage	GLH	WLH	SHG	LHG	YSB	DHB	RLF	CW	BPH	WBPH	RB	Rice Skipper	RWM
Seedling	5.69 b	2.00 a	1.46 a	0.08 c	0.31 c	0.00 d	0.46 d	0.23 c	8.46 c	4.69 ab	0.00c	0.00 c	23.46a
Active Tillering	9.51 a	1.17 b	1.51 a	0.31 a	2.16 a	0.95 a	2.45 c	0.45 a	15.4b	5.12 a	0.53 b	0.20 a	8.59 b
Maximum tillering	3.05 c	0.47 c	0.95 b	0.21 b	1.42 b	0.84 b	3.58 a	0.32 b	18.95a	4.26 b	0.78 a	0.11 b	4.47 c
Booting	1.79 d	0.32 c	0.42 c	0.26 ab	1.47 b	0.68 c	2.95 b	0.00 d	3.11 d	1.32 c	0.84a	0.11 b	1.83 d
Level of significance	∗∗∗	∗∗∗	∗∗∗	∗∗∗	∗∗∗	∗∗∗	∗∗∗	∗∗∗	∗∗∗	∗∗∗	∗∗∗	∗∗∗	∗∗∗
CV	1.35	13.20	5.01	17.71	5.56	6.97	3.53	15.62	1.53	8.35	11.84	29.71	2.49
LSD	0.13	0.25	0.10	0.72	0.14	0.08	0.16	0.07	0.33	0.61	0.12	0.06	0.45

**Table 9:** Growth stage wise abundance of beneficial insects/20 complete sweeps.

Growth Stage	SPD	Dam. fly	Drag. fly	LBB	CBB	STPD	Tiger beetle	PW	GMB
Seedling	1.15 b	0.77 c	0.08	0.38 c	0.31 c	0.31 c	0.23 bc	5.23 c	10.31 a
Active Tillering	3.71 a	1.71 a	0.16	1.04 a	1.14 a	1.14 a	0.45 a	9.31 a	3.37 b
Maximum tillering	4.00 a	1.53 a	0.11	0.63 b	1.11 a	0.47 b	0.32 b	8.95 b	3.00 b
Booting	3.95 a	1.16 b	0.16	0.37 c	0.79 b	1.05 a	0.16 c	8.84 b	1.42 c
Level of significance	∗∗∗	∗∗∗	ns	∗∗∗	∗∗∗	∗∗∗	∗∗	∗∗∗	∗∗∗
CV	6.26	9.20	36.15	9.30	9.05	5.51	20.47	1.92	4.52
LSD	0.38	0.22	–	0.11	0.14	0.08	0.11	0.29	0.39

**Footnote**, GLH: Green leafhopper; WLH: White leafhopper; SHG: Short-horned grasshopper; LHG: Long horned grasshopper; YSB: Yellow stem borer; DHB: Dark headed borer; RLF: Rice Leaffolder; CW: Caseworm, BPH: Brown planthopper; WBPH: White-backed planthopper; RB: Rice bug; RWM: Rice whorl maggot.

**Footnote**, SPD: Spider; Dam fly: Damsel fly; Drag fly: Dragon fly; LBB: Ladybird beetle; CBB: Carabid beetle; STPD: Styphylinid beetle; PW: Parasitic wasps; GMB: green mirid bug.

### 3.5 Correlation among the weather parameters, harmful and beneficial insects

**Fig. 2:**
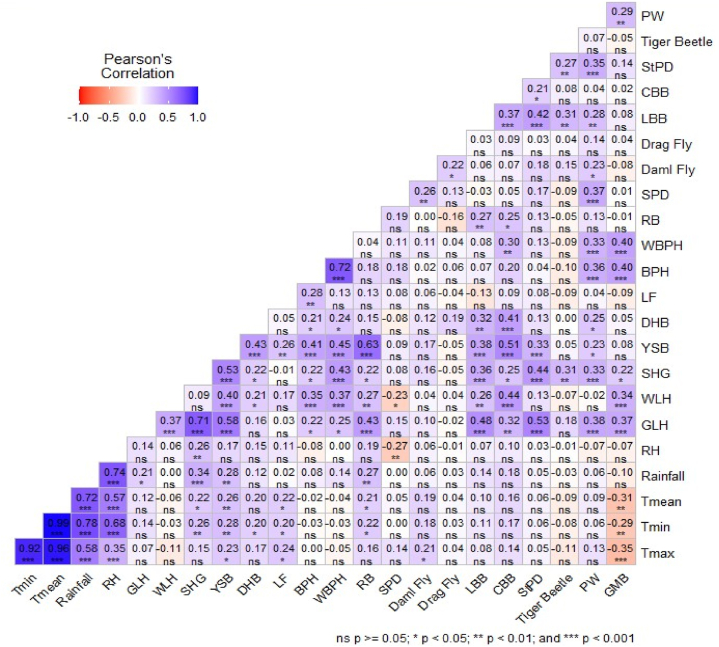
Correlation among harmful and beneficial insects and weather parameters. **Footnote**, T_max_: Maximum temperature; T_min_: Minimum temperature; T_mean_: Mean temperature;, RH: Relative humidity; GLH: Green leafhopper; WLH: White leafhopper; SHG: Short-horned grasshopper; LHG: Long horned grasshopper; YSB: Yellow stem borer; DHB: Dark headed borer; LF: Leaffolder; CW: Caseworm; BPH: Brown planthopper; WBPH: White-backed planthopper; RB: Rice bug; RWM: Rice whorl maggot; SPD: Spider; Damp fly: Damsel fly; Drag fly: Dragon fly; LBB: Ladybird beetle; CBB: Carabid beetle; StPD: Styphylinid beetle; PW: Parasitic wasps; GMB: green mirid bug.

Maximum temperature significantly influence the presence of damselfly, leaffolder and yellow stem borer. Minimum temperature has a positive and significant influence on the abundance of rice bug, leaffolder, dark headed borer, yellow stem borer, and short-horned grasshopper. On the other-hands, mean temperature positively and significantly affects the prevalence of rice bug, leaffolder, yellow stem borer, and short horned grasshopper. However, maximum temperature, minimum temperature, and mean temperature showed non-significance and negative influence on the abundance of tiger beetle, WBPH, and WLH. Conversely, minimum temperature and mean temperature was very weak non-significance and negatively correlated with the abundance of BPH. Maximum, minimum, and mean temperatures negatively and significantly influence the abundance of green mirid bug. Rainfall significantly influence the presence of rice bug, yellow stem borer, short horned grasshopper, and green leafhopper (Fig. 2). Conversely, the green mirid bug and tiger beetle showed a negative and non-significant correlation with rainfall. Relative humidity has a positive and significant influence on the presence of short horned grasshopper. A negative and significant correlation exists between relative humidity and spiders. Relative humidity negatively and non-significantly influences the presence of the green mirid bug, parasitic wasp, tiger beetle, dragonfly, and brown planthopper. The presence of the green mirid bug was significantly associated with the presence of the green leafhopper, white leafhopper, short-horned grasshopper, brown planthopper, white-backed planthopper, and parasitic wasps. Similarly, the presence of parasitic wasps was influenced by the presence of the green leafhopper, short-horned grasshopper, yellow stem borer, dark-headed borer, brown planthopper, white-backed planthopper, spider, damselfly, ladybird beetle, and staphylinid beetle. The presence of the tiger beetle was significantly influenced by the short-horned grasshopper, ladybird beetle, and staphylinid beetle.

Moreover, the presence of the styphilinid beetle was significantly associated with the presence of the green leafhopper, short horned grasshopper, yellow stem borer, ladybird beetle, and carabid beetle. In addition, carabid beetle presence was significantly influenced by the presence of the green leafhopper, white leafhopper, short horned grasshopper, yellow stem borer, dark headed borer, white-backed planthopper, rice bug, and ladybird beetle. Similarly, the presence of the ladybird beetle was significantly influenced by the presence of green leafhopper, white leafhopper, short horned grasshopper, yellow stem borer, dark headed borer, and rice bug. Significant association were found between dragon fly and damsel fly, damsel fly and spider, leaffolder and yellow stem borer, short horned grasshopper and green leafhopper, white leafhopper and green leafhopper. Moreover, the presence of the rice bug was significantly correlated with the presence of green leafhopper, white leafhopper, short horned grasshopper, and yellow stem borer. Similarly, the presence of the white-backed planthopper was significantly associated with the presence of green leafhopper, white leafhopper, short horned grasshopper, yellow stem borer, dark headed borer, and brown planthopper. On the other-hand, the presence of brown planthopper was significantly associated with the presence of green leafhopper, white leafhopper, short horned grasshopper, and yellow stem borer, dark headed borer, and leaffolder. The presence of dark headed borer was significantly associated with the presence of white leafhopper, short horned grasshopper, and yellow stem borer. Similarly, the presence of yellow stem borer was significantly associated with the presence of green leafhopper, white leafhopper, and short horned grasshopper.

## 4 Discussion

In the context of agricultural ecosystems, understanding the dynamics between harmful insects and their natural enemies is pivotal for devising effective pest management strategies. The Shannon-Wiener diversity index (H′) serves as a robust metric to gauge the biodiversity within a community. A value of 2.493 for H′, showcases a rich tapestry of both harmful and beneficial insects coexisting within the region. Remarkably, this value approaches 81 % of the maximum potential diversity (Hmax = 3.091). Shannon-Weiner diversity index for biological species assemblages typically doesn't surpass a value of 5 [30], in the majority of ecological evaluations, it tends to fall between 1.5 and 3.5 [31]. Such proximity to the maximum potential suggests that the insect community in the Rangpur division is thriving and exhibiting a commendable level of diversity. This high level of diversity can be indicative of a stable ecosystem, where various species have found their niche, ensuring ecological resilience. Furthermore, the calculated evenness of species (E) at 0.807, underscores the equilibrium in the distribution of individual insects across different species. This balanced distribution implies that no single species is overwhelmingly dominant, fostering a more harmonious coexistence among the various insect species.

The month wise distribution of harmful and beneficial insects reveals distinct temporal patterns. Harmful insects like GLH, BPH, WBPH, and RWM dominated consistently, with GLH, SHG, and RB peaking in June–July. The abundance of GLH, and RB was reached peak from April to June and from October to November and abundance of BPH, and WBPH reached peak in March to July and December [7]. WLH abundance surged in January, July, and December. YSB showed peaks from May–July and September–October, while DHB dominated February and June–October. The abundance of LF was pronounced from March–April, June and August–October, peaking in September [7]. Leaffolder reached peak population from mid September to mid October [7]. From Table 4, Table 2 it may be concluded that March to July and September to October was the most favorable month for the prevalence of most of the harmful insects and consequently higher temperature and higher (%) of relative humidity may be responsible for the higher prevalence. The abundance of most harmful insects predominantly favor the months of March and April [32]. Conversely, beneficial insects like damselfly, PW, and GMB were prevalent year-round, with SPD peaking March, June. LBB, CBB, and Styphylinid beetles exhibited specific peak months, emphasizing the intricate seasonal dynamics of insect populations.

Weather data were presented Table 2, based on weather data and month-wise distribution of insects, it was observed that the abundance of harmful insects was increased from March to April (Table 10). On that time, temperatures became increased and the difference between the maximum and minimum temperatures was narrowed. The warm weather observed from March to April stimulate insects, ending their hibernation and prompting their migration to grasses and rice fields for the initial breeding phase. Subsequently, following this first breeding cycle, insects migrate to rice fields around May to June, aligning with the period immediately after rice seedling transplantation in Aus season, and maximum tillering and booting to heading stage in Boro season (Table 1). Factor such as temperature variations, daylight duration, and the developmental stage of host plants play pivotal roles in triggering insect diapause and influencing their behaviors [33]. Temperature plays a pivotal role in fecundity, growth, development, mortality and life cycle of insects, thereby exerting a direct impact on the overall insect population [7,34]. It was observed that a 10 °C rise in temperature nearly doubles the metabolic rates of insects, subsequently boosting their fecundity, consumption, mobility, and overall population size [35]. However, findings from a study encompassing 1100 insect species suggest that the effects of global warming could lead to the extinction of approximately 15–37 % of these species by the year 2050 [35].

**Table 10:** The peak activity period of some major rice insects from January 2021 to December 2022.

Name of the insects	Month	Temperature (^0^C)	Relative Humidity (%)
Yellow Stem Borer	Jun > Jul > Sep = May	Maximum 32°C, Minimum 23°C, Optimum 26–32°C	83 %
Dark Headed Borer	Jul > Feb = Oct > Jun > Aug	Maximum 32°C, Minimum 12°C, Optimum 26–32°C	70–80 %
Brown Plant Hopper	May = Jun > Mar > Jul	Maximum 31°C, Minimum 18°C, Optimum 26–32°C	80–82 %
White Backed Plant Hopper	Jun > Dec > May	Maximum 31°C, Minimum 13°C, Optimum 26–31°C	71–80 %
Rice Leaf Folder	Sep > Jun = Mar > Oct	Maximum 31°C, Minimum 18°C, Optimum 25–31°C	82 %
Green Leaf Hopper	Jun > Jul > Dec	Maximum 31°C, Minimum 13°C, Optimum 25–31°C	79–82 %

The intensified rice farming practices in the studied region likely contribute to the increased insect populations during the transitional phases of the rice-growing season. The transition from Boro to Aus corresponds to Bangladesh's dry season, and the escalating temperatures during this period promote a more pronounced insect abundance compared to the transition from Aus to Aman season. Given that the transition from Aus to Aman coincides with the wet season, the heightened rainfall during this phase likely suppresses the insect populations [32]. Among three rice growing season, highest abundance of insects were observed in Aus season followed by T. Aman season and Boro season respectively, except BPH, WBPH and leaffolder. The abundance of leaffolder was highest in Aman season may be due to thermal condition and relative humidity [7] also reported same findings. Temperature varies from 25°C to 31°C on leaffolder peak time (Table 2, Table 10). For survival and multiplication of leaffolder 25°C–30°C temperature is favorable reported by [36]. Moreover, the fluctuations in seasonal weather conditions play a pivotal role in determining the insect presence within the agricultural fields [37]. Both Aus and T. aman seasons represent the peak of the rice cultivation period in Bangladesh, characterized by warmer temperatures conducive to insect growth and proliferation. Consequently, these two seasons record the highest insect counts. On the other-hands, Aus was not cultivated in massive area like Boro and T. Aman season, and so vast populations of insects attacked in limited areas of Aus cultivation field in Rangpur division (Fig. 1). Moreover, insects of Boro field also migrate to Aus field after Boro harvesting. Conversely, the Boro season, synonymous with Bangladesh's winter, witnesses lower temperatures and misty conditions, which inhibit insect wing functionality and impede their growth and maturation [32] therefor responsible for lower abundance of insects in Boro season. The abundance of most of the harmful insects were increased from active tillering stage followed maximum tillering stage and then gradually decreased. Green leafhoppers are found across all rice farming systems and growing stage in Bangladesh [7]. The prevalence of white leafhoppers and rice whorl maggot were highest in seedling stage then gradually decreased may be due to the softness of the seedlings. The abundance of BPH, and WBPH was increased over time and decreased at booting to heading stage. Conversely, leaffolder abundance increased over time and slightly decrease at booting to heading stage but rice bug abundance was highest at heading to booting stage. The abundance of leaffolder and rice bug were peaked at reproductive phrase [7]. Moreover, the abundance of insects were reduced in latter stage may be due to the use of insecticides. The farmers normally used 2 to 3 times insecticide in rice field after transplanting and thus reduced insect populations initially. [38] reported that the farmers of Bangladesh used 3 to 5 times insecticides on their cultivated crops.

The observed correlations between various climatic factors and insect abundance shed light on the intricate relationships within the agroecosystem. Notably, temperature variables exhibit diverse impacts on insect populations. Maximum, minimum, and mean temperatures, while positively influencing certain pests like the leaffolder and yellow stem borer, exert a negative influence on others like the tiger beetle. Such variability underscores the complexity of insect responses to temperature fluctuation. Rainfall emerges as a significant factor, particularly favoring the prevalence of pests such as the rice bug [7].

The prevalence of beneficial insects were significantly associated with the prevalence of harmful insects. Beneficial insects feed the egg, larvae as well as adults of harmful insects [37] and might be for that reason the availability of beneficial insects increased by increased with harmful insects. Green mirid bug feed on hoppers like BPH, WBPH, GLH, and SHG [7]. Parasitic wasp parasitized the egg of GLH, SHG, YSB, DHB, BPH, and WBPH. Staphylinid beetle feed on GLH, SHG, and YSB. Both adult and larvae of ladybird beetle feed on BPH, WBPH, GLH, WLH [32], and small larvae and exposed egg of YSB, DHB. The adults of carabid beetle predates GLH, BPH [32], WBPH, WLH, SHG, and larvae of YSB, DHB, RB and LF [37]. Spiders possess significant mobility, ability to quickly colonize in newly cultivated rice fields. Both adult spiders and their young prey on various harmful insects, such as the moths responsible for the rice yellow stem borer and the nymphs of plant-hoppers and leaf-hoppers [32]. Wolf spiders, in particular, consume between 5 and 15 prey daily, playing a crucial role in curtailing the population of harmful insects during the initial growth stages of rice plants [37]. The presence of certain predators or pests often corresponds with the presence of others, suggesting mutual dependencies or shared habitat preferences. Such intricate relationships underscore the need for holistic pest management strategies that consider the broader ecological context. Understanding these relationships is pivotal for devising effective, sustainable pest control measures that mitigate crop damage while preserving ecosystem balance.

## 5 Conclusion

The intricate interplay between harmful and beneficial insects within the agricultural landscape of the Rangpur division, Bangladesh, showed valuable insights into the complex dynamics of insect populations and their interactions with climatic variables. The high Shannon-Wiener diversity index value suggests a resilient insect community, indicative of a stable ecosystem of that ecosystems. Our findings illuminate distinct temporal patterns in insect prevalence, with certain months exhibiting heightened activity of harmful insects like GLH, YSB, BPH, and WBPH, while beneficial insects such as damselflies, parasitic wasps and green mirid bug maintain a consistent presence throughout the year. The abundance of harmful insects during specific rice growing seasons underscores the need for pest management strategies tailored to the unique challenges posed by each season. Weather parameters, notably temperature, RH and rainfall, exert significant influences on insect populations, with some species thriving under specific climatic conditions while others insects population declines. Rising temperatures, coupled with other weather factors, accelerate insect activity and growth, potentially intensifying pest-related challenges for rice cultivation. Conversely, lower temperatures, such as those in the Boro season, inhibit insect proliferation. The observed correlations between climatic factors and insect abundance highlight the multifaceted nature of agro ecosystem dynamics, necessitating a nuanced approach to pest management that integrates ecological considerations. Furthermore, the strong associations observed between beneficial and harmful insects emphasize the pivotal role of natural predators and parasitoids in regulating pest populations. Harnessing the potential of these biological control agents could offer sustainable alternatives to chemical insecticides, thereby reducing environmental impacts and safeguarding human health. In summary, the comprehensive analysis presented herein underscores the importance of adopting an integrated approach to pest management that encompasses ecological, climatic, and biological dimensions. Future research endeavors should focus on elucidating the underlying mechanisms driving these relationships and exploring innovative strategies to enhance the effectiveness of biological control measures in mitigating pest infestations.

## Data availability

All data accessed and analyzed in this study are available in the article and data will be made available on request.

## CRediT authorship contribution statement

**Tapon Kumar Roy:** Writing – review & editing, Writing – original draft, Supervision, Investigation, Formal analysis, Data curation, Conceptualization. **Mir Md Moniruzzaman Kabir:** Writing – review & editing, Supervision, Conceptualization. **Sanjida Akter:** Writing – review & editing, Writing – original draft, Formal analysis, Data curation. **Abu Nayeem:** Writing – review & editing, Writing – original draft, Data curation. **Zakaria Alam:** Writing – review & editing, Formal analysis, Data curation. **Md Rokebul Hasan:** Writing – review & editing, Supervision, Conceptualization. **Md Nazmul Bari:** Writing – review & editing, Supervision, Conceptualization. **Anamika Sannal:** Writing – review & editing, Writing – original draft, Supervision, Formal analysis, Data curation.

## Declaration of competing interest

The authors declare that they have no known competing financial interests or personal relationships that could have appeared to influence the work reported in this paper.
